# Improving Eye Donation Discussions in a Hospice Care Setting: A Quality Improvement Initiative

**DOI:** 10.7759/cureus.99200

**Published:** 2025-12-14

**Authors:** Euan MacInnes

**Affiliations:** 1 Anatomy, Brighton and Sussex Medical School, Brighton, GBR

**Keywords:** corneal donation, corneal transplant, deceased organ donation, eye donation, hospice and palliative care

## Abstract

Purpose: There is a critical shortage of corneal tissue in the United Kingdom (UK), limiting access to life-changing treatment for individuals with visual impairment. There are many potential donors who are admitted to a hospice setting. At our hospice, eye donation discussions do occur with some patients, but not all. This quality improvement project (QIP) aimed to evaluate and increase the frequency and quality of eye donation discussions happening in the hospice setting.

Methods: A baseline audit was conducted over one month to assess the frequency, timing, and documentation of eye donation discussions among hospice admissions. An educational intervention comprising four teaching sessions, capturing 21 inpatient unit staff members, was implemented. A post-intervention re-audit was conducted over the following month.

Results: In the baseline audit, 14 patients were admitted; seven were eligible for eye donation, and only three (43%) were asked about donation. Only one discussion occurred at the time of admission, and the reason for exclusion was documented for just two of the seven ineligible patients. Post-intervention, 25 patients were admitted, 21 were eligible, and 11 (52%) were asked about donation. Discussion at the time of admission increased from 33% to 64% and documentation of ineligibility improved from 29% to 100%.

Conclusions: The educational intervention improved staff engagement and documentation related to eye donation, with a modest increase in discussion frequency, but a substantial increase in exclusion documentation. However, cultural and timing barriers persist. Continued education and integration of donation discussions into standard workflows are recommended for sustained improvement.

## Introduction

Eye donation plays a critical role in improving and often restoring the vision of people with visual impairment due to corneal disease by providing corneal tissue for transplantation [1]. Despite the exclusion criteria being less strict than other organ donations, such as solid organ, due to the avascular nature of the cornea [2] and the relatively straightforward procedure of eye retrieval, there remains a significant shortage of donor eye tissue both nationally and globally [3]. 

Eye donation does not have to satisfy the same criteria that solid organs must comply with. Where solid cancers are an exclusion criterion for solid organ transplantation, this is not the case for eye donation. Due to the immune privilege experienced by the eye, as well as the avascular nature of the cornea, it lends itself well to transplantation and does not require the need for lifelong immunosuppressants [4]. 

Eye donation can occur up to 24 hours postmortem [5]. This allows time for the coordination of the donation to be arranged, without the rush and high financial burden of operating theatres and life support machines that are required for a solid organ transplant. These two factors combined make a hospice an ideal setting for eye donation to occur. Furthermore, corneas can be stored for up to 28 days following harvest, allowing ample time for return to the eye bank [6]. 

Hospice care is patient-centred and holistic in nature. It is designed to allow time for informed discussions and choices regarding death and dying. The hospice offers a calm and respectful setting that many other healthcare settings do not offer. This allows healthcare professionals to delve into often difficult subject areas, such as eye donation, which people often find more difficult to talk to about due to the personal nature of eyes compared to solid organs like the liver [2]. Furthermore, allowing patients and families the opportunity to explore eye donation may provide comfort to the family in a final altruistic act. 

Despite its potential, eye donation is infrequently discussed in a hospice setting. A study looking at hospice patients in three initiations over the course of three years found that around 50% of service users were eligible to donate eye tissue. Despite this, only 4% of patients were approached to discuss eye donation [2]. 

There is a massive shortage of eye tissue in the United Kingdom (UK). In England, more than 4,000 people are living with sight loss that could be restored or significantly improved with a corneal transplant [7]. The weekly target for eye donation in the UK is 350, but only around 88 per week are achieved [2]. Furthermore, one pair of donated eyes can be used in up to 10 sight-altering operations, highlighting the impact just one donation can make [1]. 

To meet the demand for donor eye tissue, an appropriate first step is to increase patients' awareness of the subject, explain the potential benefits and the process of the procedure, and ask the patients if they would like to participate. This audit aimed to increase the number of eye donation discussions taking place at St Wilfrid's Hospice in Eastbourne, England.

## Materials and methods

The quality improvement project (QIP) was conducted at St Wilfrid's Hospice in southern England. The only criterion for inclusion was that patients must be admitted to our hospice as inpatients. This could be for end-of-life care or for palliative rehabilitation. All inpatients admitted to our hospice over a one-month period had their electronic patient records retrospectively examined for evidence of patients being asked if they would like to take part in eye donation following death. Records were also examined for outcomes of eye donation discussions, at which point during admission eye donation discussions took place, and if not eligible to donate, a documented reason for exclusion. 

We conducted two cycles using a plan-do-study-act (PDSA) model, with an educational intervention consisting of four in-person didactic teaching sessions delivered over a period of five days to a total of 21 members of the hospice clinical ward team. Inpatient staff members attending the teaching sessions mainly consisted of nursing and medical staff. The teaching sessions lasted approximately 30 minutes and were followed by a question-and-answer session. The teaching sessions covered: how donated eye tissue is used, the conditions it can be used to treat, the outcome of corneal transplant for people living with corneal disease, and the impact on the quality of life of recipients of corneal tissue. Each teaching session was the same session delivered four times.

The teaching was well-received with positive feedback. The month following these teaching sessions was then retrospectively audited in the same fashion as cycle 1. 

## Results

All patients admitted to the hospice were screened over two one-month periods (cycle 1 and cycle 2). There were 14 admissions during cycle 1 and 25 admissions during cycle 2 (Table 1). By examining patient notes, we assessed evidence of eye donation discussions that took place, at what point during the admission these discussions took place, whether patients agreed to donate, and any documented reason for exclusion, if applicable (Table 2).

**Table 1:** Patients admitted and eligibility to donate eye tissue across both cycles.

Question	Cycle 1	Cycle 2
Patients admitted to the hospice, n	14	25
Patients eligible to donate eyes, n	7	21

**Table 2:** Retrospective results of both audit cycles.

Question	Cycle 1	Cycle 2
Eligible patients who were asked if they would like to donate eye tissue, n (%)	3 (43%)	11 (52%)
Eligible patients who were asked during the initial assessment (day 1 of admission), n (%)	1 (33%)	7 (64%)
Eligible patients agreeing to the donation of eye tissue, n (%)	2 (66%)	6 (55%)
Patients with a documented reason for exclusion, n (%)	2 (29%)	4 (100%)

## Discussion

Following the educational intervention, there was an improvement in the frequency of eye donation discussions taking place in our hospice. Notably, these discussions were not only happening more frequently but also occurring earlier in the patient's admission. In audit cycle 2, 64% of patients who were approached regarding eye donation had the conversation during the initial assessment on day 1 of admission, compared to only 33% in audit cycle 1. 

A common topic of discussion during the delivery of the educational intervention was the “right time” to initiate discussions around eye donation. It became clear that clinical staff felt the most appropriate time was during the initial assessment. By asking on day 1, the discussion can be incorporated into many other conversations and feel less intimidating, creating a more natural time to bring up the subject. Additionally, it addresses the concern of raising the topic later - particularly during a change in a patient's condition or phase of illness - which some members of the team found to be an inappropriate time that may cause distress to the patient or family. However, it was also highlighted that the initial assessment is already a busy and emotionally heavy time for the patient, and waiting may be appropriate if the discussion could potentially overwhelm them. 

Documentation of exclusion improved following the intervention. Documenting exclusion is relevant as it ensures all members of the clinical team are aware of their eye donation status. It avoids unnecessary conversations, which could lead to disappointment and frustration, especially if a patient has expressed a wish to donate eye tissue and then is subsequently told they will not be able to do so. It also ensures transparency that eye donation has been actively considered rather than passively avoided. 

A study by Madi-Segwagwe et al. [8] conducted a scoping review aimed at identifying barriers and facilitators to eye donation in a hospice setting. They found 13 articles focusing on this topic. Similar to this QIP, they found reluctance among healthcare professionals to bring up the subject due to the intimate and emotional burden the topic entails. They also identified a lack of awareness of donation options and eligibility, which was something not explored in this QIP. Furthermore, they highlighted the lack of clinical guidelines available to guide eye donation conversations. 

In a study by Long-Sutehall et al. [9], a survey of palliative care and hospice care clinicians evaluated the attitudes and practices regarding eye donation in the end-of-life care setting. The survey revealed that although many clinicians supported the idea of eye donation, it was rarely part of routine end-of-life planning. This somewhat differs from the findings of this QIP, where eye donations were already occurring semi-frequently. This highlights our hospice’s efforts to introduce eye donation discussions prior to this QIP. Furthermore, this study once again highlighted the lack of standardized policy around eye donation in the hospice setting. 

Sample size was a significant limiting factor in this QIP. With data collected over the course of only two months, there remains considerable scope for improvement with longer data collection periods in future cycles of the QIP. Furthermore, this limitation reduces the generalizability of the study.

## Conclusions

This audit aimed to evaluate the frequency, timing, and documentation of eye donation discussions taking place in a hospice setting, as well as to identify opportunities for improvement. Following an educational intervention, the frequency of eye donation discussions increased, along with the proportion of these discussions occurring during the initial admission assessment. Furthermore, clear documentation of exclusions improved, reflecting more thoughtful and active note-keeping practices. However, due to the small sample size and single-centre nature of the study, generalizability may be limited. Additionally, there remains a proportion of patients who are eligible for eye donation but are not being given the opportunity to discuss their wishes. Through continued staff education and a planned further audit cycle, we hope to give every patient in our hospital the opportunity to discuss their wishes regarding eye donation.
